# 1757. Regional distribution of *Escherichia coli* antibiotic resistance among outpatients in Washington state, 2013-2019

**DOI:** 10.1093/ofid/ofac492.1387

**Published:** 2022-12-15

**Authors:** Hannah T Fenelon, Peter M Rabinowitz, Ann Salm, Hema Kapoor, Stephen E Hawes, Jeff Radcliff

**Affiliations:** University of Washington, Seattle, Washington; University of Washington, Seattle, Washington; Quest Diagnostics, Secaucus, New Jersey; Quest Diagnostics, Secaucus, New Jersey; University of Washington, Seattle, Washington; Quest Diagnostics, Secaucus, New Jersey

## Abstract

**Background:**

*Escherichia coli* is the predominant pathogen of urinary tract infections in the United States. Understanding regional patterns of uropathogenic *E. coli* antimicrobial resistance (AMR) may help further the practice of antibiotic stewardship through the creation of region-specific antibiograms. In this study, we analyzed regional patterns of uropathogenic *E. coli* AMR among outpatients in Washington state.

**Methods:**

Deidentified results from antibiotic susceptibility tests performed by Quest Diagnostics on Washington state outpatient isolates from 2013 through 2019 were analyzed. Only the first *E. coli* isolate from each patient was included in the analyses. We conducted separate relative risk regressions with robust standard errors for five antibiotics (ampicillin [AMP], ciprofloxacin [CIP], ceftriaxone [CRO], gentamicin [GEN], and trimethoprim/sulfamethoxazole), classified as “susceptible” or “non-susceptible.” The state is divided into nine Public Health Emergency Preparedness Regions (PHEPRs), which served as the exposures for the analysis, with adjustment for sex, year of isolate collection, and age group (0-18, 19-50, > 50).

**Results:**

We included 40,865 isolates in the study (93% female, mean age 47 years). Compared to the Central PHEPR (containing Seattle), most other regions had significantly lower adjusted relative risks (aRR) of AMR; no regions had significantly higher aRR of resistance for any of the five antibiotics. Differences in resistance between Central and other regions varied by antibiotic. Regional AMR differences were largest for CRO (low aRRs compared to Central) and smallest for AMP. Other patterns observed include a lower aRR of AMR for females compared to males for AMP, CIP, CRO, and GEN; higher aRR of AMR for CRO for recent years (2015-2019) compared to 2013; and higher AMR with age for CIP and CRO.

**Conclusion:**

Given the differences in resistance across Washington’s PHEPRs, antibiograms should be tailored to provide the most regionally accurate information to prescribers. The emergent situation surrounding AMR calls for more specific outpatient antibiograms to enable healthcare providers to improve health outcomes and mitigate emerging resistance to antibiotics among outpatients.

**Disclosures:**

**Ann Salm, PhD**, Quest Diagnostics Inc.: Employed and own stock in Quest Diagnostics Inc.|Quest Diagnostics Inc.: Stocks/Bonds **Hema Kapoor, MD, D(ABMM)**, Quest Diagnsotics: Stocks/Bonds **Stephen E. Hawes, PhD**, InBios International: Advisor/Consultant **Jeff Radcliff, B.S.**, Quest Diagnostics: Employee|Quest Diagnostics: Stocks/Bonds.

